# Supplementary material for: Comparative Genomic Analysis of PEBP Genes in Cucurbits Explores the Interactors of Cucumber CsPEBPs Related to Flowering Time
Source: Int J Mol Sci. 2024 Mar 29;25(7):3815. doi: 10.3390/ijms25073815 (PMC11011414; doi:10.3390/ijms25073815)
Supplement: Supplementary file 1 [file ijms-25-03815-s001.zip › Figure S2.pdf]

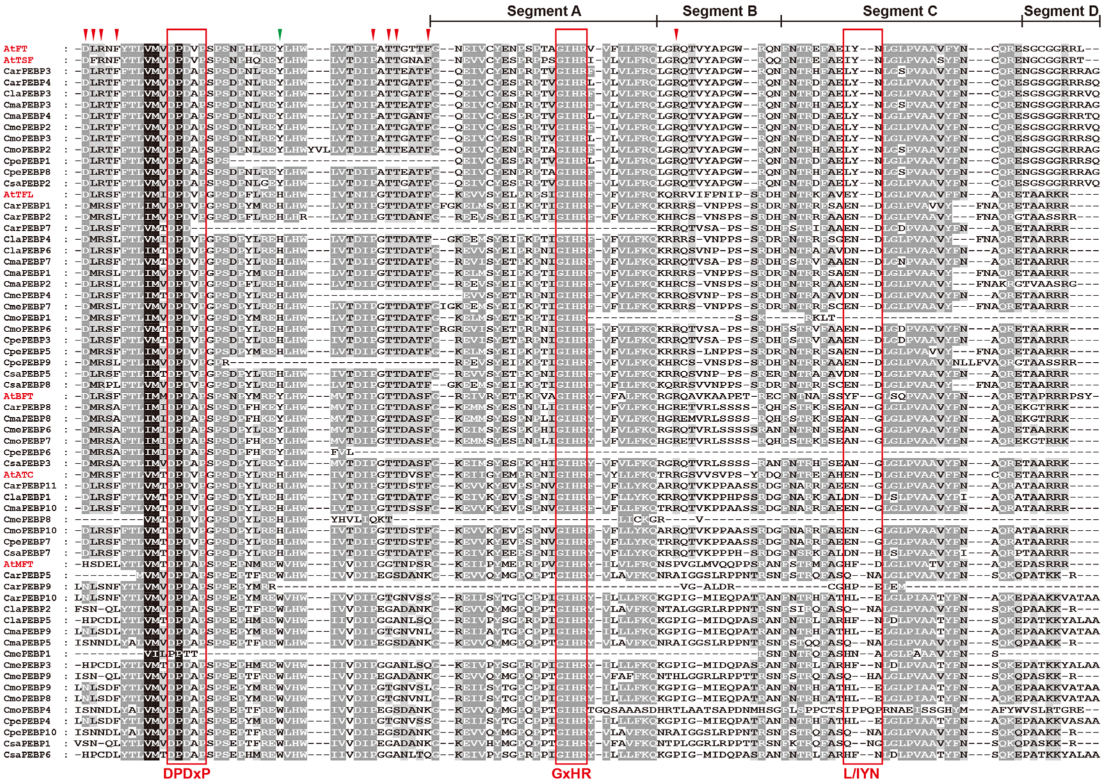

Figure S2. Comparative analysis of PEBP proteins from the MFT, FT and TFL clades between Arabidopsis and seven cucurbit crops. Red triangle indicates amino acid residues that interact with 14-3-3 protein. The green triangle indicates a key amino acid residue that determines MFT-like, FT-like and TFL-like proteins. Red boxes represent the conserved DPDxP, GxHR motif and L/IYN. Underlines represent segments A, B, C and D, respectively.
