# Supplementary material for: Comparative Genomic Analysis of PEBP Genes in Cucurbits Explores the Interactors of Cucumber CsPEBPs Related to Flowering Time
Source: Int J Mol Sci. 2024 Mar 29;25(7):3815. doi: 10.3390/ijms25073815 (PMC11011414; doi:10.3390/ijms25073815)
Supplement: Supplementary file 1 [file ijms-25-03815-s001.zip › Figure S3.pdf]

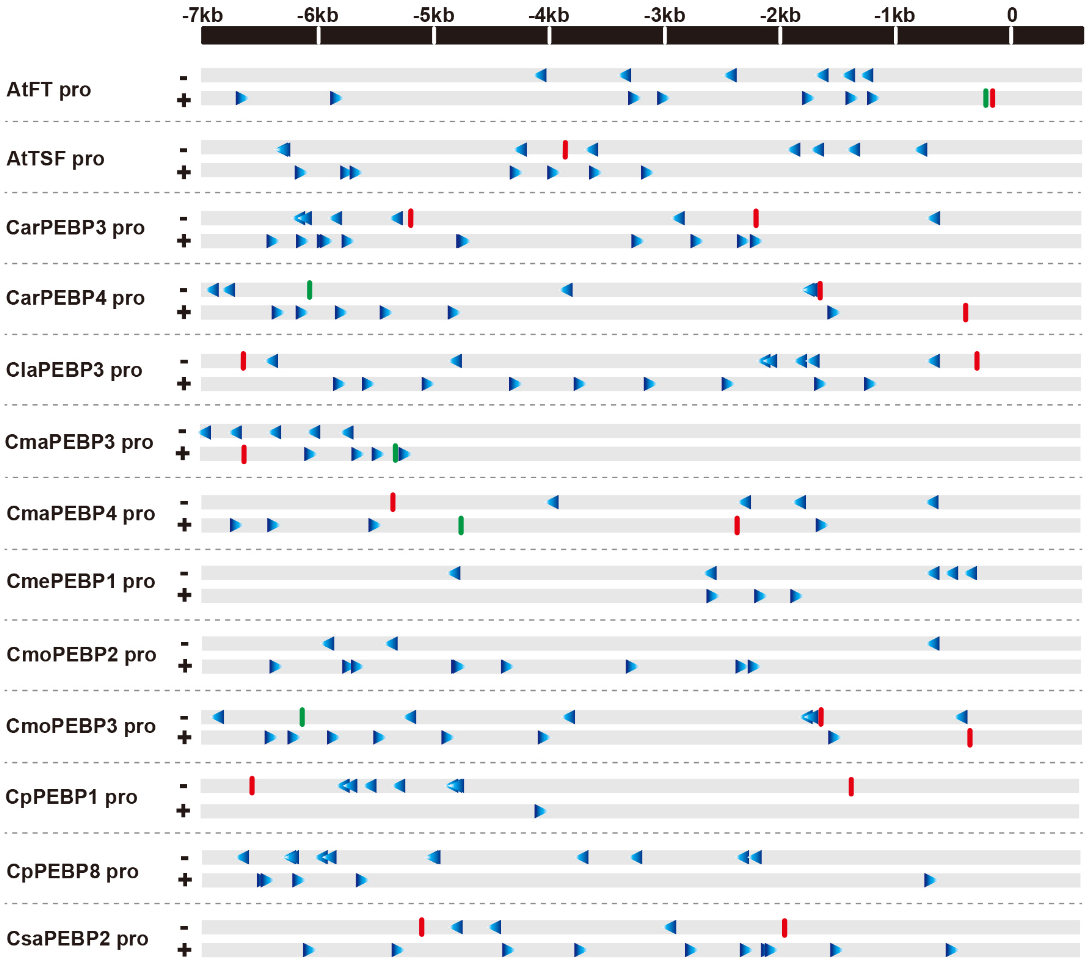

Figure S3. Scale diagram of the 7000 bp promoter regions of the cucurbit *FT/TSF-like* gene. The blue triangles represent CCAAT boxes. Green and red lines indicate CORE1 and CORE2 regions, respectively.
