# Supplementary figures and images for: Comparative Genomic Analysis of PEBP Genes in Cucurbits Explores the Interactors of Cucumber CsPEBPs Related to Flowering Time
Source: Int J Mol Sci. 2024 Mar 29;25(7):3815. doi: 10.3390/ijms25073815 (PMC11011414; doi:10.3390/ijms25073815)

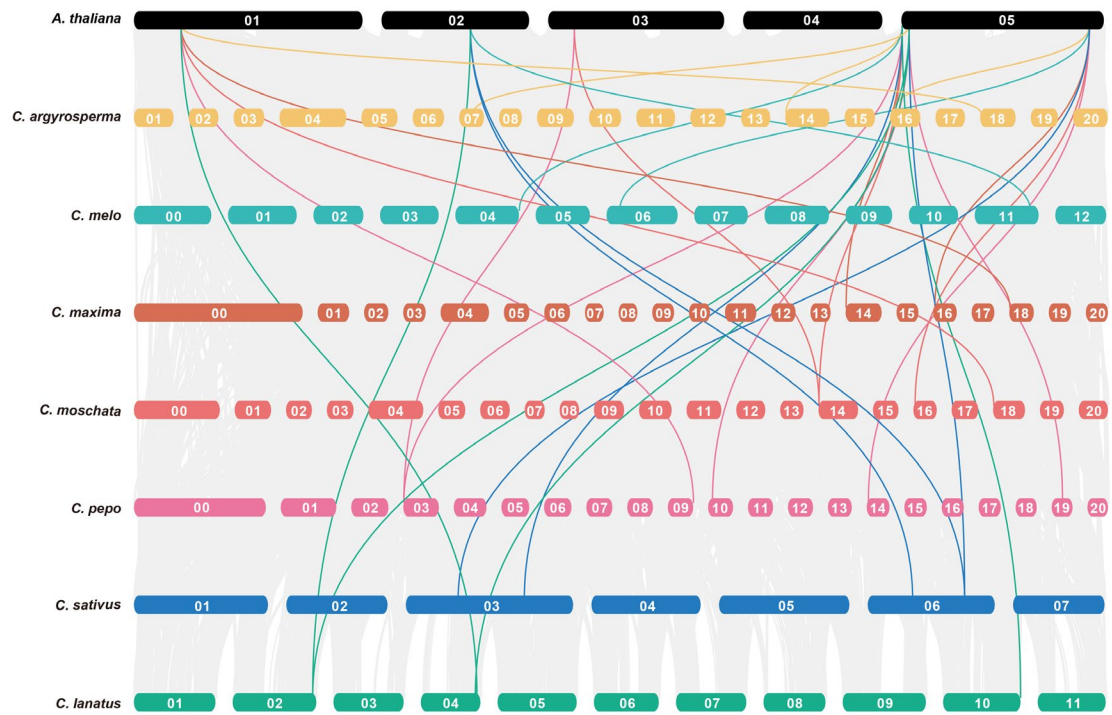

**Figure S1.** A syntentic map of PEBP genes between *Arabidopsis* and seven cucurbit crops.

Supplement: Supplementary file 1 [file ijms-25-03815-s001.zip › Figure S1.pdf]
